# Nanoparticles in Thyroid Autoimmunity: Diagnostic and Therapeutic Applications

**DOI:** 10.3390/jcm15041428

**Published:** 2026-02-12

**Authors:** Giusy Elia, Silvia Martina Ferrari, Francesca Ragusa, Eugenia Balestri, Chiara Botrini, Federica Colapietra, Paola Della Monica, Alessandro Antonelli, Poupak Fallahi, Marina Di Domenico

**Affiliations:** 1 Department of Surgical, Medical and Molecular Pathology and Critical Area, University of Pisa, 56126 Pisa, Italy; giusy.elia@unipi.it (G.E.); silvia.ferrari@unipi.it (S.M.F.);; 2 Department of Precision Medicine, University of Campania Luigi Vanvitelli, Via Abramo Lincoln, 5, 81100 Caserta, Italy; 3 Department of Clinical and Experimental Medicine, University of Foggia, Via A. Gramsci 89/91, 71100 Foggia, Italy; 4 Department of Translational Research and New Technologies in Medicine and Surgery, University of Pisa, 56126 Pisa, Italy

**Keywords:** nanoparticles, thyroid autoimmunity, nanodiagnosis, nanotherapy, autoimmune thyroiditis, Graves’ disease, thyroid eye disease

## Abstract

Autoimmune thyroid diseases (AITDs) represent T cell-mediated, organ-specific autoimmune disorders caused by immune dysregulation, culminating in an immune-mediated attack on thyroid tissue. AITD etiopathogenesis is the result of the interplay between a genetic susceptibility and environmental factors; hypothyroidism and thyrotoxicosis are the respective clinical hallmarks of autoimmune thyroiditis and Graves’disease, the two main forms of AITD. The application of nanomedicine in the context of thyroid disorders ranges from nanodiagnosis and nanotherapy to nanotheranostics. Nanomedicine has been used to develop new sensitive methods for the determination of the TSH, iodine and TSAb. Furthermore, other studies have used nanomedicine to explore new treatments of autoimmune thyroiditis, Graves’disease and also thyroid eye disease. In the future, the application of nanomedicine will be personalized in accordance with individual genetic profiles, thus improving the therapeutic effectiveness and reducing the undesirable side effects with improved patient outcomes.

## 1. Introduction

The nanoparticles (NPs) are those materials whose dimensions are in the nanoscale range (1–100 nm) [1,2]. They are classified as inorganic, carbon-based, or organic, and are further differentiated in metallic, ceramic, polymeric, and lipid-based, according to their specific characteristics, size and shape [3,4].

Inorganic NPs can be synthesized from semiconductors, ceramics, or magnetic metals and represent one of the most commercially used common NP classes, particularly for anticancer, antioxidant and antibacterial purposes [3,5].

Organic NPs are made of proteins, lipids, polymers, and carbohydrates; they exhibit low toxicity and are biodegradable. Thanks to these characteristics, organic NPs are widely used with many different approaches, such as targeted drug delivery, bioimaging, cancer therapy, and biosensing [6]. Among organic NPs, lipid-based systems are the most extensively studied and widely used for the delivery of immunotherapeutic agents [7]. These systems include liposomes, lipid NPs, solid lipid NPs, nanostructured lipid carriers, nano-emulsions, and hybrid lipid NPs [8,9].

Carbon-based NPs, including graphene, fullerenes, and carbon black NPs, exhibit high chemical stability, superior thermal and electrical conductivity, strong optical absorption, and luminescence [10], which allow their use in biosensing, drug delivery, and cancer and cell therapies, as well as in in vivo, in vitro, and optical imaging [11]. These different properties enable NPs to be employed in multiple applications, such as catalysis, imaging, biomedical and energy-associated research, and environmental applications [12].

NPs hold an immense potential for cancer diagnosis and therapy; when they enter into contact with biological fluid, such as blood, they interact with biomolecules and acquire a biomolecular shell. This shell is called biomolecular corona, whose composition depends on several factors, including the features of NPs, the characteristics of biological fluids (e.g., protein origin and concentration, temperature), and the time of incubation [13,14,15,16,17,18,19,20,21]. Moreover, the multifunctionality and tailorability of NPs make them excellent as drug delivery systems. Therefore, the field of nanomedicine allows significant opportunities for therapeutics and diagnostic applications.

We have conducted a narrative review by reviewing the scientific literature about NPs in thyroid autoimmunity. We have searched on PubMed for the literature about NPs in thyroid autoimmunity by using keywords such as “nanoparticles in autoimmunity”, “nanoparticles in autoimmune thyroid diseases”, “nanoparticles in thyroid autoimmunity”, “nanoparticles in Graves’ disease”, “nanoparticles in hypothyroidism”, “nanoparticles in thyroid eye disease”, “nanoparticles in hyperthyroidism”, “nanoparticles in autoimmune thyroiditis”, “nanoparticles in Graves’ ophthalmopathy”.

### 1.1. Nanoparticles Immunomodulation

A therapeutic approach against autoimmune diseases, or cancer and other inflammatory-related disorders, exploits immunomodulation, which allows one to intentionally activate or inhibit the immune system.

In this scenario, NPs can be useful for a specific targeted stimulation of the immune system by delivering immune checkpoint inhibitors (ICIs), antigens, or cytokines to modulate the immune responses [22,23,24,25,26].

Cytokine-loaded NPs have been shown to activate T and NK cells in the tumoral microenvironment, while minimizing effects on healthy tissues [27].

NPs can also encapsulate chemotherapeutic drugs and ICIs, such as anti-PD-1, a humanized IgG4 monoclonal antibody which exhibits immune checkpoint inhibitory and anticancer properties, as well as anti-CTLA-4 antibodies, another class of ICIs applied in cancer therapy [28].

By targeting tumors and modulating the immune checkpoints, NPs are able to enhance the efficacy of immunotherapy, offering a promising therapeutic option for patients who do not respond to conventional therapies.

### 1.2. Nanoparticles in Autoimmune Disease

Autoimmune diseases are a consequence of the immune system dysregulation resulting in the attack of the body instead of providing protection [29].

This leads to chronic inflammation and tissue damage, as seen in different autoimmune diseases, such as Graves’disease (GD), Hashimoto’s thyroiditis (HT), type 1 diabetes (T1D), rheumatoid arthritis (RA), inflammatory bowel disease, multiple sclerosis, and others [30].

Immunosuppressants are widely used to treat autoimmune diseases, but they may increase the risk of infections and other adverse effects [31].

New immunological approaches are exploiting the use of specific therapy such as monoclonal antibodies (mAbs) with limited systemic effects. However, therapies based on mAbs also report several disadvantages, such as antibody immunogenicity’s ability to render subsequent therapies ineffective or to be associated with toxicities (anti-IL-6R) and with susceptibility to infectious diseases [32].

The novel NPs can preserve the immune functions by promoting antigen-specific tolerance, and can improve the effect of a targeted therapy by delivering the drugs directly in the specific inflamed site. Moreover, the specific anti-inflammatory response can be modulated with designed multi-functional NPs able to respond to environmental stimuli inhibiting and/or promoting inflammation [33].

In preclinical models, a delayed onset of RA has been observed by using hybrid NPs that co-target B and T cells. This approach reduces autoantibody production and promotes the immune suppression of the regulatory T cell (Treg), with no impact on healthy tissues [34,35].

Similarly, other NPs have been synthesized to selectively target inflammation-activated macrophages overexpressing folate receptors [36]; in RA rat models, this results in an important reduction in paw volume, ankle swelling, bone resorption and cartilage damage [37].

It is also noteworthy that NPs can be engineered to respond to specific stimuli, enabling activity only under pathological conditions. In fact, the tumor microenvironment has unique characteristics, such as hypoxia, acidic pH (6.5–6.8), and upregulation of specific enzymes [38]. A specific response to disease-associated triggers allows NPs to localize to the areas where they are most needed, thereby improving effectiveness and reducing side effects [39].

### 1.3. Different Strategies for Nanotherapy in Autoimmunity

**(a)**  **Antigen target**

As reported above, autoimmune diseases are a consequence of a breakdown of immune tolerance to self-antigens; therefore, antigen-specific treatments can be developed to selectively eliminate the autoimmune responses while maintaining the protective immune responses [40].

Approaches involve the targeted elimination of autoreactive lymphocytes, tolerization of autoreactive T cells, and active elimination of autoimmune responses. Along with vaccines, cell-based therapies, and gene-editing strategies, NPs represent some of the most promising tools for these purposes.

NPs can be designed to encapsulate immunomodulatory agents such as small molecules, peptides, nucleic acids, or biologics, and can be subsequently directed to antigen-presenting cells (APCs) through surface modifications or passive uptake, facilitating the transfer of the therapeutic payload precisely to immune cells [41].

Moreover, antigens can be combined with modulators of co-stimulation or immune response, such as immunomodulatory small molecules, cytokines, or other biologic components. Lipid-based or liposome NPs can encapsulate lipophilic immunomodulatory agents and be functionalized to target a tolerogenic environment, or to specific APCs via receptor-specific ligands or antibodies. Biodegradable drug delivery particles are preferred thanks to their safety profile and for their ability to enable localized or targeted drug delivery. In contrast, metal NPs, e.g., iron or gold, may be associated with toxicities or allergy. Immunomodulatory agents encompass NF-κB and/or mTOR inhibitors, including rapamycin, calcitriol, the aryl-hydrocarbon receptor (AhR) ligand 2-(1′H-indole-3-carbonyl)-thiazole-4-carboxylic acid methyl ester, curcumin and TGF-β1, as well as co-inhibitory molecules that are able to stimulate regulatory T cells, like PD-L1 [42].

Antigen-specific therapy is a promising approach for autoimmune disorders treatment; nevertheless, several challenges persist, such as choosing the most suitable antigen, the optimal administration strategy and timing, and identifying the diseases in which these therapies are most beneficial.

A double-blind study for celiac disease has investigated the use of poly (dl-lactide-co-glycolic acid) (PLGA) to encapsulate gluten protein to achieve targeted gluten tolerance [43], showing that this technology could be a promising approach in comparison with other conventional treatments [44].

**(b)**  **Immunomodulator delivery**

An innovative approach for treating autoimmune diseases utilizes mRNA-lipid NPs. This platform allows a variety of therapeutic strategies, such as targeted modulation of immune cells, antigen-specific tolerizing immunotherapy, and in vivo chimeric antigen receptor (CAR) T cell therapies [45].

Naked mRNA is highly vulnerable to nuclease degradation during its systemic transfer en route to the target site. Lipid NPs can therefore act as vehicles to enable precise, efficient and safe mRNA administration while supporting durable immune responses [46,47,48].

Clinical trials involving exosome-mimetic NPs (EMNPs) are emerging, with most studies focusing on their diagnostic and therapeutic potential in inflammatory conditions.

A phase I/II clinical trial is investigating EMNPs loaded with anti-inflammatory miRNAs for the treatment of inflammatory bowel disease [49]; in addition, another study is exploring EMNPs delivering IL-10 mRNA to macrophages in Crohn’s disease patients [50]. EMNPs delivering tolerogenic myelin oligodendrocyte glycoprotein (MOG) peptides are under evaluation in a phase I trial for patients with multiple sclerosis [44].

NPs can serve as controlled drug delivery platforms, allowing precise spatial and temporal dosing to promote antigen-specific tolerance in autoimmune diseases. Controlled immunomodulation through these systems enables targeted, localized immune regulation in chronic conditions, reducing off-target effects, enhancing therapeutic efficacy, and reducing toxicities [51]. A spatiotemporally controlled release can be obtained through systems that leverage pH, reactive oxygen species (ROS), or enzyme-responsive mechanisms, for example, by confining TNF-α siRNA release to inflamed joints in RA [52].

Several clinical studies on RA and SLE patients are evaluating NPs combined with different drugs (such as methotrexate (MTX) or dexamethasone) and engineered to respond to specific stimuli (ROS, pH) [44,53,54,55].

**(c)**  **Checkpoint regulation/modulation of co-inhibitory signaling**

The PD-1/PD-L1 immune checkpoint plays a central role in maintaining peripheral tolerance by inhibiting autoreactive T cells. Unlike traditional therapies like mAbs or checkpoint ligands, nanocarrier-based approaches, ranging from liposomes or lipid NPs to polymeric NPs, biomimetic cell membrane-coated vehicles and extracellular vesicles (EVs), allow localized and controlled activation of immune checkpoints for targeted delivery of PD-1/PD-L1 agonists.

These strategies allow the combined delivery of tolerogenic agents and optimize biodistribution while reducing toxicity at systemic levels [56]. Polymeric NPs can function as versatile platforms, capable of delivering PD-1/PD-L1-activating molecules or presenting PD-L1 ligands on their surface to inflamed tissues.

A novel strategy involves hybrid NPs co-loaded with MTX and surface-functionalized with recombinant PD-L1. These NPs can selectively inhibit CD4+ and CD8+ T cells by binding PD-1 on their surface, while promoting Treg production [57]. Moreover, MTX helps in reducing systemic inflammation and lymphocyte activation. Recent studies are also investigating liposomal systems for the co-delivery of PD-1 pathway activators together with other immune–modulatory peptides or RNAs [58], such as liposomes loaded with siRNAs targeting PD-L1-suppressive miRNAs in dendritic cells (DCs), resulting in upregulating endogenous PD-L1 expression on DCs within target tissues.

## 2. Thyroid Autoimmunity

### 2.1. Autoimmune Thyroid Disorders

Autoimmune thyroid diseases (AITDs) represent T cell-mediated, organ-specific autoimmune disorders caused by immune dysregulation, culminating in an immune-mediated attack on thyroid tissue. AITD etiopathogenesis is the result of the interplay between a genetic susceptibility and environmental factors; hypothyroidism and thyrotoxicosis are the respective clinical hallmarks of autoimmune thyroiditis (AT) and GD, the two main forms of AITD [59,60].

Diffuse lymphocytic infiltration of the thyroid, mainly composed of thyroid-specific T and B cells, together with elevated serum levels of autoantibodies against one or more thyroid antigens, are hallmark features of both AT and GD [61,62,63]. Thyroid follicular cell atrophy and fibrosis are key characteristics of thyroiditis, whereas the hypertrophied thyroid follicles are found in GD [64]. Nonetheless, AITD can develop even without detectable circulating antithyroid autoantibodies [65].

Cytokines and chemokines play a central role in the pathogenesis of AT and GD. In thyroid tissue, Th1 lymphocytes promote IFN-γ and TNF-α production, which in turn stimulates CXCL10 secretion by thyroid cells [59]. Mainly CXCL10, the prototype IFN-γ–inducible T helper 1 (Th1) chemokine, contributes to the initiation and perpetuation of the autoimmune process. It has been hypothesized that CXCL10 is a marker of a more aggressive inflammatory response in the thyroid, leading to lymphocyte infiltration, thyroid destruction and hypothyroidism. CXCL10 acts by binding with the chemokine (C-X-C motif) receptor 3 (CXCR3) [66] and is involved in the development of various autoimmune disorders, including organ-specific T1D, GD, and thyroid eye disease (TED), as well as systemic lupus erythematosus, Sjögren’s syndrome, mixed cryoglobulinemia, psoriasis, sarcoidosis, and systemic sclerosis [67,68,69,70,71,72]. Patients with AT have elevated circulating CXCL10 levels, especially in combination with a hypoechoic ultrasonographic pattern, indicative of more pronounced lympho-monocytic infiltration, as well as in patients with hypothyroidism [73]. Moreover, CXCL10 also plays a role in the development of GD and TED; so, under the stimulation of IFN-γ, CXCL10 is produced by thyrocytes in GD, as well as by fibroblasts and preadipocytes in TED [74,75,76].

### 2.2. Nanoparticles in Thyroid Autoimmunity

Thyroid diseases, including HT and hypothyroidism, GD and hyperthyroidism, are considered among the most prevalent autoimmune endocrine disorders that remain diagnostically challenging. Recently, many innovations in nanomedicine, such as nanotechnology-based rapid and portable diagnostic devices and targeted delivery of pharmaceutics for thyroid disorders, have shown significant promise [77]. The application of nanomedicine in the context of thyroid disorders ranges from nanodiagnosis and nanotherapy to nanotheranostics (Table 1).

**(a)**  **Nanoparticles for diagnosis of thyroid dysfunction**

The thyroid-stimulating hormone (TSH) is a biomarker of thyroid function, that is used widely for monitoring thyroid function, as well as for the evaluation of therapeutic efficacy and to assess the medication dosing. TSH measurements are particularly susceptible to immunoassay interference caused by autoantibodies present in the sample matrix. Therefore, new immunoassays with a higher precision and sensitivity are under evaluation, such as those utilizing NPs.

A super-sensitive TSH immunoassay was developed by Näreoja et al., by using NP labels, with a detection limit of 60 nU L−1 in serum samples, while minimizing non-specific binding [78]. This assay reported an enhanced sensitivity attributed to the stabilization of the protein corona of the NP bioconjugates and to a spot-coated configuration of the active solid phase. This effect minimized the sedimentation of the NP bioconjugates and reduced their time of contact with antibody-coated solid phase, exploiting simultaneously the higher association rate of specific binding provided by the high avidity NP bioconjugates [78].

Iodine is essential for thyroid hormone biosynthesis and plays a fundamental role in fetal nervous system development [90]. In this context, a nanotechnology biosensor was developed to measure the iodine levels in urine samples. This methodology was demonstrated to be fast, specific, and sensitive, capable of detecting various disorders resulting from iodine imbalance, including hyperthyroidism [79].

Hypercalcemia, resulting from hyperthyroidism, may contribute to dementia, muscle weakness and osteoporosis. To address this, a bioinspired gold NP-based sensing system was designed, by using calsequestrin, the main diffuse calcium-binding protein, to detect abnormal serum Ca2+levels, offering a potential promising tool for the detection and monitoring of hyperthyroidism [80].

Other studies explored the use of NPs for the detection of autoantibodies to TSH receptor stimulating autoantiodies (TSAbs), that are pathognomonic for the diagnosis of GD. Sugamata et al. showed a system in which recombinant hTSHR was expressed on magnetic NPs’ lipid-bilayer surface obtained from a magnetostatic bacterium. This system proved to be helpful for the analysis of the interaction between ligand and receptor or for TSAb immunoassay in patients with GD [81].

**(b)**  **Nanoparticles in thyroiditis and for the treatment of hypothyroidism**

The etiopathogenesis of AITD is the result of the combination of genetic predisposition, environmental factor interaction, and oxidative stress. In this context, because of the antioxidant properties of selenoproteins, a Selenium supplementation could be useful for both AT and GD disorders [91]. Selenium NPs (SeNPs) are very promising due to their high permeability and low or no toxicity, and they could be successfully used in the targeted delivery of drugs, such as anti-inflammatory or anticancer agents [92]. SeNPs enter into cells through endocytosis and are able to deliver differently charged macromolecules and antitumor agents at a nanoscale [93].

A dual-action polymeric system was developed for the treatment of hypothyroidism. The system releases Cepharanthine to inhibit T cell activation and Selenium to reduce anti-thyroid peroxidase (TPOAb) levels, thereby providing a treatment for hypothyroidism. The dual-delivery system was shown to be effective for the long-term treatment of hypothyroidism and is able to target both the autoimmune response and hormone regulation [82].

A therapeutic approach using CTLA4Ig/silica-NPs was employed for the treatment of experimentally induced canine autoimmune thyroiditis [83]. The authors reported a marked reduction in anti-canine-thyroglobulin autoantibody titer, anti-T4 antibody titer and T cell proliferation in response to thyroglobulin, as well a reduction in interleukin-18 mRNA in freshly peripheral blood mononuclear cells (PBMCs) obtained from dogs. They also demonstrated an important reduction in INF-γ or TNF-α cytokine levels in cultured PBMCs. CTLA4Ig appeared to restore Th1/Th2 cytokine balance in autoimmune thyroiditis through the downregulation of Th1 cytokines [83].

**(c)**  **Nanoparticles in Graves’disease**

In GD, antigen presentation is dysregulated, with a reduction in tolerogenic DCs and abnormal expression of MHC class II by non-professional follicular cells [42,94,95,96]. The immune response is characterized by an early Th1-immune predominance that shifted to a chronic Th2-immune predominance [97]. Immunosuppressive therapies, including glucocorticoids, azathioprine, rituximab, cyclosporine and tocilizumab have been studied in GD—however, with a constrained use due to their significant related adverse effects [98].

GD represents a promising model for tolerogenic antigen-specific immunotherapy (ASI). The regulation of the autoimmune response could maintain the integrity of thyroid endocrine function and achieve long-term remission. Nevertheless, thyrotropin receptor (TSHR) antigenic epitopes responsible for GD development are still not completely understood. A phase 1 trial studied ATX-GD-59, an antigen-specific immunotherapy with a combination of two TSHR peptides. This open-label, uncontrolled study enrolled 12 patients with HLA-DR3 or DR4, from mild to moderate GD, not previously treated with anti-thyroid drugs, and administered escalating intradermal doses of ATX-GD-59 [99]. The treatment was well-tolerated, and at the end of the study, 7 out of 10 participants reported improved thyroid function, 5 out of 10 reduced antibody levels, 8 out of 10 remained subclinically hyperthyroid, and 2 required anti-thyroid therapy. However, in the absence of a control group, the clinical relevance is uncertain, as spontaneous remission occurs in 10–25% of cases [100]. This study highlights the potential of ASI in GD and opens the way for future antigen-specific immunotherapy strategies.

DC-targeted therapies aimed at inducing antigen-specific immune tolerance represent a promising strategy for GD. Polylactic-co-glycolic acid (PLGA) NPs were developed to encapsulate the GD autoantigen TSHR-A peptide, together with the immune tolerance inducer rapamycin. This construct was proved to be efficiently internalized by DCs in in vitro studies, whereas in in vivo studies it was observed to be accumulated in the mice spleens. The NPs inhibit maturation of DCs in vitro and in vivo and also modulate the secretion of cellular functional factors from DCs. Furthermore, they lead to the proliferation of Treg cells in vivo and in vitro. In addition, NPs were able to prevent and mitigate disease progression in mouse models without adverse side effects at the organ level. Therefore, PLGA-based NP encapsulating (TSHR-A+rapamycin) represents a promising DC-targeted method in order to modulate immune response for a new potential therapeutic approach to GD [84].

A thyroid-targeted nano-bomb system (PSAPI) was designed to enhance high-intensity focused ultrasound (HIFU) efficacy and improve therapeutic outcomes for GD. The core structure of PSAPI contains a phase-transition material, perfluorohexane, and the anti-inflammatory drug diclofenac within a PLGA and silica shell. The presence of the polydopamine coating improves biocompatibility, while iodine incorporation and TRAB antibody functionalization allows the targeted delivery to the thyroid. PSAPI proved to be highly biocompatible and capable of accumulating mostly in the thyroid, with a significant enhancement of HIFU therapeutic efficacy, leading to markedly reduced inflammatory responses [85].

**(d)**  **Nanoparticles in Thyroid Eye Disease**

TED is an autoimmune disorder of the orbit characterized by proptosis, inflammation and extraocular muscle inflammation that affects about 30–50% of patients with GD. Mild forms of TED are treated with local measures (eye drops or Selenium). Moderate to severe forms of TED are traditionally treated with intravenous corticosteroids, orbital radiotherapy or surgery. More recently targeted therapies have been developed, such as Teprotumumab (a fully human monoclonal antibody against the IGF1-receptor), that has been shown to be effective in reducing inflammation, proptosis and diplopia [101]. However, more efforts are needed to find more effective treatments of TED.

Cerium oxide NPs (CNPs) are emerging in the ophthalmology research, because of their anti-inflammatory and antifibrotic potential. CNPs were studied on orbital fibroblast (OF) in patients with TED by Wang et al. [86]. They demonstrated that CNPs suppress the generation of ROS by OF, and down-regulated the expression of fibrosis markers. CNPs also decreased the expression of mRNA and of the inflammatory cytokines IL-6 and TNF-α [86].

Exosomes obtained from the tear fluid (TF) of TED patients and their exosomal protein content were analyzed in comparison with controls [87]. TF exosome quantity was determined by NP tracking analysis. Control OFs were then cultured in the presence of TF exosomes obtained from patients with TED or controls, to evaluate their influence on the expression of inflammatory cytokines. Exosomes isolated in TF from TED patients were more numerous than those from controls, and moreover, the levels of exosomal proteins, such as vitamin D-binding protein, chitinase 3-like 1, C-reactive protein and vascular cell adhesion molecule-1, were significantly increased in patients with TED with respect to controls. OFs treated with TF exosomes from TED patients reported a significantly increased production of IL-8, IL-6, and CC motif chemokine ligand 2 (CCL2) compared to OFs treated with TF exosomes from controls. Moreover, certain proteins were present at high concentrations in exosomes from TED patients, suggesting their potential roles in the TED pathogenesis [87].

In another study, the authors explored the role of exosomes in TED, by enrolling 24 TED patients, 24 GD patients and 16 controls. The quantity of tear exosomes was measured by using NPs tracking analysis. IL-1 and IL-18 levels were significantly higher both in serum and in tear exosomes obtained from TED patients in comparison with GD or healthy controls. Levels of other proteins, such as complement C4A, APOA-IV and Caspase-3, were higher in exosomes derived from TED in comparison with GD and controls. These results demonstrated that the expression of some specific proteins was upregulated in tear exosomes from TED patients, highlighting their crucial role in TED pathogenesis [88].

Emerging studies have highlighted the involvement of mitochondria-associated endoplasmic reticulum membranes (MAMs) in the pathogenesis of TED [89]. MAMs can contribute to TED pathogenesis trough different mechanisms, including: (a) disruption of calcium homeostasis with a subsequent activation of NADPH oxidase and the increase in ROS production [102]; (b) dysregulation of lipid metabolism, that leads to an adipose tissue proliferation [103]; and (c) amplification of pro-inflammatory signaling pathways (activation of NLRP3 inflammasome and release of mitochondrial DNA) resulting in increased levels of proinflammatory cytokines such as IL-1β [104]. A dual-responsive Selenium NP (Se@LNT) modified with lentinan, and designed for ROS/pH-triggered release, was investigated in human primary OF, and its therapeutic mechanism was studied in TED mouse models. Se@LNT represents the first nanotherapeutic strategy targeting MAMs for TED by modulating the disease progression at the subcellular level. Se@LNT, through GPX1-mediated restoration of MAM function, reverses adipose hyperplasia in TED. Furthermore, Se@LNT has dual regulatory effects by rebalancing the immune responses and inhibiting the PPARG/CEBPA-driven adipogenic network. These results suggest a Selenium-based nanotherapeutic strategy for TED [89].

## 3. Nanoparticles in Thyroid Cancer

In late 1863, Virchow was the first to suggest a relation between chronic inflammation and cancerogenesis [105].

Further studies have also assessed a link between AITD and an increasing incidence of thyroid cancer (TC); indeed, thyroid autoimmunity and elevated TSH levels were defined as independent risk factors for TC [106].

TC is one of the most common endocrine malignancies that comprises four histological variants, namely follicular, papillary, anaplastic deriving from the follicular cells and medullary cancers arising from para-follicular C cells of neuroendocrine origin [107].

Current conventional therapies include surgery, radioiodine therapy, chemotherapy, targeted therapy and immunotherapy on the basis of tumor histotype. The multiple applications of NPs make them suitable for diagnosis as well as for delivery of therapeutic drugs to the specific tumor tissue.

NPs represent a future personalized therapeutic approach able to enhance 131I sensitivity and chemotherapy, to improve drug solubility and to reduce immunogenicity. However, more studies are needed to further investigate the interaction between NP target cells and tissues. An important issue to be faced is the monitoring of the drug release kinetics for a better and safe patient outcome. Hybrid nanoparticles, thanks to the combination of different NP properties (organic, inorganic, and targeting), allow the use of this system for different clinical applications, such as drug/gene delivery, laser-assisted therapies, and imaging.

Therefore, hybrid NPs improve diagnosis and the control of the drug release by enhancing their accumulation in the tumor, resulting in a better efficacy [108].

## 4. Nanoparticles: Limitations and Possible Toxicity

NPs represent an innovative approach for the treatment of autoimmune diseases and cancer; however, several limitations are still present.

A primary limit is due to the potential toxicity of nanomaterials, particularly metal-based NPs such as silver or titanium dioxide that can generate ROS, inducing oxidative stress and inflammation in tissues, thus leading to long-term health risks, including organ damage or carcinogenesis by damaging cells, DNA, and proteins [44].

A relevant aspect related to NP toxicity has been associated with their capabilities to set themselves all around the protein by forming a sort of “protein corona”, where the stabilization depends on particle size, shape and other specific characteristics. This binding is able to induce adverse effects on both protein structure and biological activity [109].

Another limitation is caused by the failure in reaching the tissue-specific target, resulting in toxicity and an off-target accumulation in non-diseased tissues.

Functionalizing NPs with highly targeted ligands (antibodies or aptamers) could improve tissue specificity [22].

Along with toxicity NP clearance is another important challenge to face, in this context, self-destructing NPs, that disassemble post-delivery, are being actively investigated to achieve a safe elimination from the body.

Furthermore, biomimetic coatings (derived from cell membranes) could be a strategic approach to lower a possible NP-related immunogenicity [22].

## 5. Conclusions

Nanomedicines is an emerging and very promising field in thyroid dysfunctions. Clinically, liposomes and LNPs have demonstrated well-established success [1,110].

In 1995, Doxil (liposomal doxorubicin) was the first approved nanodrug by the Food and Drug Administration (FDA). Subsequently, a small interfering ribonucleic acid (siRNA) encapsulated in LNPs (Onpattro) was approved in 2018 by the FDA for amyloidosis [111,112]. Lipid-based NP drugs have also been recently approved by the FDA and European Medicines Agency for specific immunological applications, for example, Mepact, a liposome formulated with the immunomodulatory peptide-lipid mifamurtide, and a nucleotide-binding oligomerization domain containing 2 (NOD2)-agonist, that activated monocytes and macrophages against tumor cells [113], and Comirnaty and Spikevax, the LNP-mRNA vaccines developed for COVID-19 [114].

The research application of NPs is very encouraging, spanning from infectious diseases [115,116] to autoimmune diseases [117] and cancer [118].

Nanomedicine has been used to develop new sensitive methods for the determination of the TSH, iodine and TSAb. Furthermore, other studies have used nanomedicine to explore new treatments for autoimmune thyroiditis, Graves’ disease and also thyroid eye disease. In the future, the application of nanomedicine will be personalized in accordance with individual genetic profiles, thus improving the therapeutic effectiveness and reducing the undesirable side effects with improved patient outcomes.

## Figures and Tables

**Table 1:** Nanoparticles in thyroid autoimmunity.

	Studies	References
**Nanoparticles for diagnosis of thyroid dysfunction**	A super-sensitive immunoassay for TSH detection (NPs labels, with a detection limit of 60 nU L−1 in serum samples).	**Näreoja T et al. [78]**
	Nanotechnology biosensor developed to measure iodine levels in urine samples for detection of various disorders from iodine imbalance, including hyperthyroidism.	**Ibrahim NH et al. [79]**
	Detection of serum Ca^2+^ levels using calsequestrin-functionalized gold NPs.	**Kim S et al. [80]**
	Detection of TSAb with NPs expressing recombinant hTSHR on lipid-bilayer surface from a magnetostatic bacterium.	**Sugamata Y et al. [81]**
**Nanoparticles for the treatment of hypothyroidism**	A dual-delivery system releases Cepharanthine and Selenium for hypothyroidism treatment.	**Kutlu Kaya C et al. [82]**
	CTLA4Ig/silica-NPs employed for the treatment of experimentally induced canine autoimmune thyroiditis.	**Choi EW et al. [83]**
**Nanoparticles in Graves’ disease**	PLGA-based NP encapsulating (TSHR-A+rapamycin) as DC-targeted therapies to induce antigen-specific immune tolerance.	**Chen K et al. [84]**
	PSAPI containing a phase-transition material, perfluorohexane, and the anti-inflammatory drug diclofenac within a PLGA and silica shell to enhance high-intensity focused ultrasound efficacy and improve therapeutic outcomes for GD.	**Wang B et al. [85]**
**Nanoparticles in Thyroid Eye Disease**	CNPs suppress the generation of ROS by OF, and down-regulated the expression of fibrosis markers.	**Wang BW et al. [86]**
	Evaluation of tear exosomes in TED patients. The number of exosomes and the levels of exosomal proteins (e.g., vitamin D-binding protein, chi-tinase 3-like 1, C-reactive protein and vascular cell adhesion molecule-1) were significantly increased in patients with TED with respect to controls.	**Han JS et al. [87]**
	Evaluation of tear exosomes in TED patients. IL-1 and IL-18 levels were significantly higher both in serum and tear exosomes from TED patients in comparison with GD or healthy controls. Also, other proteins (complement C4A, APOA-IV and Caspase-3) were higher in exosomes derived from TED in comparison with GD and controls.	**Shi TT et al. [88]**
	Investigation of dual-responsive Selenium NPs (Se@LNT) modified with lentinan, and designed for ROS/pH-triggered release, in human primary OF, and evaluation of its therapeutic mechanism in TED mouse models. Se@LNT represents the first nanotherapeutic strategy targeting MAMs for TED by modulating the disease progression at the subcellular level.	**Tan Y et al. [89]**

**Abbreviations:** CNPs: Cerium oxide NPs; hTSHR: human thyrotropin receptor (TSHR); MAMs: mitochondria-associated endoplasmic reticulum membranes; NPs: nanoparticles; OF: orbital fibroblast; PLGA: Polylactic-co-glycolic acid; PSAPI: thyroid-targeted nano-bomb system; ROS: reactive oxygen species; TSAb: TSH receptor stimulating autoantibodies; TSH: thyrotropin.

## Data Availability

No new data were created or analyzed in this study.

## References

[1] Cheng Z., Fobian S.F., Gurrieri E., Amin M., D’Agostino V.G., Falahati M., Zalba S., Debets R., Garrido M.J., Saeed M. (2024). Lipid-based nanosystems: The next generation of cancer immune therapy. J. Hematol. Oncol..

[2] Mulvaney P. (2015). Nanoscience versus nanotechnology: Defining the field. ACS Nano.

[3] Eker F., Duman H., Akdaşçi E., Bolat E., Sarıtaş S., Karav S., Witkowska A.M. (2024). A Comprehensive Review of Nanoparticles: From Classification to Application and Toxicity. Molecules.

[4] Ijaz I., Gilani E., Nazir A., Bukhari A. (2020). Detail Review on Chemical, Physical and Green Synthesis, Classification, Characterizations and Applications of Nanoparticles. Green. Chem. Lett. Rev..

[5] Kim T., Hyeon T. (2014). Applications of Inorganic Nanoparticles as Therapeutic Agents. Nanotechnology.

[6] Mitragotri S., Stayton P. (2014). Organic Nanoparticles for Drug Delivery and Imaging. MRS Bull..

[7] Zhang Z., Yao S., Hu Y., Zhao X., Lee R.J. (2022). Application of lipid-based nanoparticles in cancer immunotherapy. Front. Immunol..

[8] Tenchov R., Bird R., Curtze A.E., Zhou Q. (2021). Lipid nanoparticles: From liposomes to mRNA vaccine delivery, a landscape of research diversity and advancement. ACS Nano.

[9] Mukherjee S., Ray S., Thakur R.S. (2009). Solid lipid nanoparticles: A modern formulation approach in drug delivery system. Indian J. Pharm. Sci..

[10] Kokorina A.A., Ermakov A.V., Abramova A.M., Goryacheva I.Y., Sukhorukov G.B. (2020). Carbon Nanoparticles and Materials on Their Basis. Colloids Interfaces.

[11] Singh R.P., Singh K.R.B. (2021). Nanobiotechnology in Animal Production and Health. Advances in Animal Genomics.

[12] Khan I., Saeed K., Khan I. (2019). Nanoparticles: Properties, applications and toxicities. Arab. J. Chem..

[13] Tenzer S., Docter D., Rosfa S., Wlodarski A., Kuharev J., Rekik A., Knauer S.K., Bantz C., Nawroth T., Bier C. (2011). Nanoparticle size is a critical physicochemical determinant of the human blood plasma corona: A comprehensive quantitative proteomic analysis. ACS Nano.

[14] Walkey C.D., Olsen J.B., Guo H., Emili A., Chan W.C.W. (2012). Nanoparticle size and surface chemistry determine serum protein adsorption and macrophage uptake. Am. Chem. Soc..

[15] Caracciolo G., Pozzi D., Capriotti A.L., Cavaliere C., Foglia P., Amenitsch H., Laganà A. (2011). Evolution of the protein corona of lipid gene vectors as a function of plasma concentration. Langmuir.

[16] Monopoli M.P., Walczyk D., Campbell A., Elia G., Lynch I., Baldelli Bombelli F., Dawson K.A. (2011). Physical−chemical aspects of protein corona: Relevance to in vitro and in vivo biological impacts of nanoparticles. J. Am. Chem. Soc..

[17] Caracciolo G., Pozzi D., Capriotti A.L., Cavaliere C., Piovesana S., La Barbera G., Amici A., Laganà A. (2014). The liposome-protein corona in mice and humans and its implications for in vivo delivery. J. Mater. Chem. B.

[18] Mahmoudi M., Abdelmonem A.M., Behzadi S., Clement J.H., Dutz S., Ejtehadi M.R., Hartmann R., Kantner K., Linne U., Maffre P. (2013). Temperature: The “ignored” factor at the NanoBio interface. ACS Nano.

[19] Barrán-Berdón A.L., Pozzi D., Caracciolo G., Capriotti A.L., Caruso G., Cavaliere C., Riccioli A., Palchetti S., Laganà A. (2013). Time evolution of nanoparticle-protein corona in human plasma: Relevance for targeted drug delivery. Langmuir.

[20] Tenzer S., Docter D., Kuharev J., Musyanovych A., Fetz V., Hecht R., Schlenk F., Fischer D., Kiouptsi K., Reinhardt C. (2013). Rapid formation of plasma protein corona critically affects nanoparticle pathophysiology. Nat. Nanotechnol..

[21] Di Domenico M., Pozzi D., Palchetti S., Digiacomo L., Iorio R., Astarita C., Fiorelli A., Pierdiluca M., Santini M., Barbarino M. (2019). Nanoparticle-biomolecular corona: A new approach for the early detection of non-small-cell lung cancer. J. Cell. Physiol..

[22] Al-Thani A.N., Jan A.G., Hajialthakar Z., Awad A., Abbas M. (2025). Next-generation nanoparticles for cancer and autoimmune therapy. Biochem. Pharmacol..

[23] Hegde M., Mishra A., Banerjee R., Bintee B., Alqahtani M.S., Abbas M., Shanmugam M.K., Drum C.L., Sethi G., Liu L. (2025). Bioresponsive engineered nanoparticles for immunomodulation. BMC Med..

[24] Barik P., Mondal S. (2025). Immunomodulatory effects of metal nanoparticles: Current trends and future prospects. Nanoscale.

[25] Liu T., Yang L., Hou Y., Tian Y., Ashrafizadeh M., Orive G., Han M., Yang Y. (2025). Programmable metal-based immuno-photocatalytic nanoparticles for precision activation of anti-tumor immunity. J. Nanobiotechnol..

[26] Piao H., Jiang Y., Jin S., Shi J., Yu J., Wang W., Du Z., Yao H., Liu Q., Li N. (2025). Modulation of the immune microenvironment using nanomaterials: A new strategy for tumor immunotherapy. Front. Immunol..

[27] Wells K., Liu T., Zhu L., Yang L. (2024). Immunomodulatory nanoparticles activate cytotoxic T cells for enhancement of the effect of cancer immunotherapy. Nanoscale.

[28] Immune Checkpoint Inhibitors—NHI. https://www.cancer.gov/about-cancer/treatment/types/immunotherapy/checkpoint-inhibitors.

[29] Nasir N., Mak A. (2025). Autoimmune Diseases. Int. Encycl. Public Health.

[30] Strzelec M., Detka J., Mieszczak P., Sobocińska M.K., Majka M. (2023). Immunomodulation-a general review of the current state-of-the-art and new therapeutic strategies for targeting the immune system. Front. Immunol..

[31] Hussain Y., Khan H. (2022). Immunosuppressive Drugs. Encycl. Infect. Immun..

[32] Prosperi D., Colombo M., Zanoni I., Granucci F. (2017). Drug nanocarriers to treat autoimmunity and chronic inflammatory diseases. Semin. Immunol..

[33] Sagnik N., Souray M., Mohammed G.A., Vetriselvan S. (2024). “Smart” stimuli-responsive biomaterials revolutionizing the theranostic landscape of inflammatory arthritis. Mater. Today Chem..

[34] Brzezicka K.A., Arlian B.M., Wang S., Olmer M., Lotz M., Paulson J.C. (2022). Suppression of Autoimmune Rheumatoid Arthritis with Hybrid Nanoparticles That Induce B and T Cell Tolerance to Self-Antigen. ACS Nano.

[35] Nanjaiah H., Moudgil K.D. (2024). Targeted Therapy of Antibody-Induced Autoimmune Arthritis Using Peptide-Guided Nanoparticles. Int. J. Mol. Sci..

[36] Thomas T.P., Goonewardena S.N., Majoros I.J., Kotlyar A., Cao Z., Leroueil P.R., Baker J.R. (2011). Folate-targeted nanoparticles show efficacy in the treatment of inflammatory arthritis. Arthritis Rheum..

[37] Liu J., Liu Z., Pang Y., Zhou H. (2022). The interaction between nanoparticles and immune system: Application in the treatment of inflammatory diseases. J. Nanobiotechnol..

[38] Wu J., Xue X., Qu H. (2025). Tumor microenvironment-responsive nanoplatforms for enhanced cancer immunotherapy: Advances and synergistic strategies. Nano Trans. Med..

[39] Jameema Sidhic M.K.A., Prasad A., Alby T., Mohan P., Sarbadhikary P., Narayanankutty A., Satheesh G., Abrahamse H., Blassam P.G. (2025). Advancements in metal and metal oxide nanoparticles for targeted cancer therapy and imaging: Mechanisms, applications, and safety concerns. J. Drug Deliv. Sci. Technol..

[40] Buckner J.H. (2025). Antigen-specific immunotherapies for autoimmune disease. Nat. Rev. Rheumatol..

[41] Puccetti M., Costantini C., Schoubben A., Giovagnoli S., Ricci M. (2024). Strategies and delivery systems for cell-based therapy in autoimmunity. Front. Drug Deliv..

[42] Zala A., Thomas R. (2023). Antigen-specific immunotherapy to restore antigen-specific tolerance in Type 1 diabetes and Graves’ disease. Clin. Exp. Immunol..

[43] Kelly C.P., Murray J.A., Leffler D.A., Getts D.R., Bledsoe A.C., Smithson G., First M.R., Morris A., Boyne M., Elhofy A. (2021). TAK-101 Nanoparticles Induce Gluten-Specific Tolerance in Celiac Disease: A Randomized, Double-Blind, Placebo-Controlled Study. Gastroenterology.

[44] Barakat M., Abu Ershaid J.M., Alzaghari L.F., Abdulrazzaq S.B., Raad D., Hasen E., Al-Qudah R., Chellappan D.K., Athamneh T., Al-Najjar M. (2026). How Effective are Nanotechnology-Based Therapeutics to Treat Autoimmune Diseases. Int. J. Nanomed..

[45] Razavi R., Kegel M., Muscat-Rivera J., Weissman D., Melamed J.R. (2025). Harnessing mRNA-lipid nanoparticles as innovative therapies for autoimmune diseases. Mol. Ther. Methods Clin. Dev..

[46] Baden L.R., El Sahly H.M., Essink B., Kotloff K., Frey S., Novak R., Diemert D., Spector S.A., Rouphael N., Creech C.B. (2021). COVE Study Group. Efficacy and Safety of the mRNA-1273 SARS-CoV-2 Vaccine. N. Engl. J. Med..

[47] Polack F.P., Thomas S.J., Kitchin N., Absalon J., Gurtman A., Lockhart S., Perez J.L., Pérez Marc G., Moreira E.D., Zerbini C. (2020). Safety and Efficacy of the BNT162b2 mRNA Covid-19 Vaccine. N. Engl. J. Med..

[48] Goel R.R., Gouma S., Kuri-Cervantes L., Hicks P., Dysinger S., Hicks A., Sharma H., Herring S., Korte S., Baxter A.E. (2021). mRNA vaccines induce durable immune memory to SARS-CoV-2 and variants of concern. Science.

[49] De Leo V., Catucci L., Maurelli A.M., Daniello V., Conese M., Di Gioia S. (2025). Liposomes and exosome-like nanoparticles for curcumin delivery to the gut and to treat inflammatory bowel disease. Advances in Novel Phytopharmaceuticals.

[50] Bu T., Li Z., Hou Y., Sun W., Zhang R., Zhao L., Wei M., Yang G., Yuan L. (2021). Exosome-mediated delivery of inflammation-responsive Il-10 mRNA for controlled atherosclerosis treatment. Theranostics.

[51] Girela M., Gupta D., Grattoni A., Chua C.Y.X. (2026). Targeted immunomodulation for chronic diseases through advanced delivery platforms. Expert Opin. Drug Deliv..

[52] Huang W., Lu J., Xue Z., Tang B., Tian J., Yang L. (2025). Harnessing Biomaterials for Gene Therapy in Autoimmune Disease. Adv. Healthc. Mater..

[53] Chen M., Amerigos JC K.D., Su Z., Guissi N.E.I., Xiao Y., Zong L., Ping Q. (2019). Folate Receptor-Targeting and Reactive Oxygen Species-Responsive Liposomal Formulation of Methotrexate for Treatment of Rheumatoid Arthritis. Pharmaceutics.

[54] Zhu H., Huang D., Nie M., Zhao Y., Sun L. (2024). Dexamethasone loaded DNA scavenger nanogel for systemic lupus erythematosus treatment. Bioact. Mater..

[55] You Y., Zhou Z., Wang F., Li J., Liu H., Cheng X., Su Y., Chen X., Zheng H., Sun Y. (2024). Mycophenolate Mofetil and New-Onset Systemic Lupus Erythematosus: A Randomized Clinical Trial. JAMA Netw. Open.

[56] Xiang G., Cui Y., Wang P., Feng Y., Zhang C., Lou J., Zhou X. (2025). Nanomedicine targeting the PD-1/PD-L1 axis in autoimmune diseases: Breaking conventional barriers to restore immune tolerance. J. Nanobiotechnol..

[57] An E.K., Zhang W., Park H.B., Kim S.J., Eom H.Y., Hwang J., Kwak M., Lee J.Y., Lee P.C., Jin J.O. (2023). Immunosuppressive nanoparticles containing recombinant PD-L1 and methotrexate alleviate multi-organ inflammation. Biomaterials.

[58] Lin G., Wang J., Yang Y.G., Zhang Y., Sun T. (2023). Advances in dendritic cell targeting nano-delivery systems for induction of immune tolerance. Front. Bioeng. Biotechnol..

[59] Antonelli A., Ferrari S.M., Corrado A., Di Domenicantonio A., Fallahi P. (2015). Autoimmune thyroid disorders. Autoimmun. Rev..

[60] Ferrari S.M., Fallahi P., Ruffilli I., Elia G., Ragusa F., Benvenga S., Antonelli A. (2019). The association of other autoimmune diseases in patients with Graves’ disease (with or without ophthalmopathy): Review of the literature and report of a large series. Autoimmun. Rev..

[61] Paparo S.R., Ferrari S.M., Patrizio A., Elia G., Ragusa F., Botrini C., Balestri E., Guarneri F., Benvenga S., Antonelli A. (2022). Myoinositol in Autoimmune Thyroiditis. Front. Endocrinol..

[62] Romagnani S. (1998). The Th1/Th2 paradigm and allergic disorders. Allergy.

[63] Orgiazzi J. (2012). Thyroid autoimmunity. Presse Med..

[64] Armengol M.P., Juan M., Lucas-Martín A., Fernández-Figueras M.T., Jaraquemada D., Gallart T., Pujol-Borrell R. (2001). Thyroid autoimmune disease: Demonstration of thyroid antigen-specific B cells and recombination-activating gene expression in chemokine-containing active intrathyroidal germinal centers. Am. J. Pathol..

[65] Rotondi M., Coperchini F., Magri F., Chiovato L. (2014). Serum-Negative Autoimmune Thyroiditis: What’s in a Name?. J. Endocrinol. Investig..

[66] Antonelli A., Rotondi M., Fallahi P., Ferrari S.M., Paolicchi A., Romagnani P., Serio M., Ferrannini E. (2006). Increase of CXC chemokine CXCL10 and CC chemokine CCL2 serum levels in normal ageing. Cytokine.

[67] Antonelli A., Ferrari S.M., Giuggioli D., Ferrannini E., Ferri C., Fallahi P. (2014). Chemokine (C-X-C motif) ligand (CXCL)10 in autoimmune diseases. Autoimmun. Rev..

[68] Antonelli A., Baj G., Marchetti P., Fallahi P., Surico N., Pupilli C., Malvasi F., Ferrannini E. (2001). Human anti-CD38 autoantibodies raise intracellular calcium and stimulate insulin release in human pancreatic islets. Diabetes.

[69] Antonelli A., Fallahi P., Delle Sedie A., Ferrari S.M., Maccheroni M., Bombardieri S., Riente L., Ferrannini E. (2009). High values of Th1 (CXCL10) and Th2 (CCL2) chemokines in patients with psoriatic arthritis. Clin. Exp. Rheumatol..

[70] Antonelli A., Ferri C., Fallahi P., Ferrari S.M., Sebastiani M., Ferrari D., Giunti M., Frascerra S., Tolari S., Franzoni F. (2008). High values of CXCL10 Serum levels in mixed cryoglobulinemia associated with hepatitis C in- fection. Am. J. Gastroenterol..

[71] Antonelli A., Ferri C., Fallahi P., Cazzato M., Ferrari S.M., Sebastiani M., Ferrannini E. (2007). Clinical and subclinical autoimmune thyroid disorders in systemic sclerosis. Eur. J. Endocrinol..

[72] Antonelli A., Ferri C., Fallahi P., Colaci M., Giuggioli D., Ferrari S.M., Frascerra S., Franzoni F., Galetta F., Ferrannini E. (2008). Th1 and Th2 chemokine serum levels in systemic sclerosis in the presence or absence of autoimmune thyroiditis. J. Rheumatol..

[73] Ruffilli I., Ferrari S.M., Colaci M., Ferri C., Fallahi P., Antonelli A. (2014). IP-10 in autoimmune thyroiditis. Horm. Metab. Res..

[74] Antonelli A., Rotondi M., Ferrari S.M., Fallahi P., Romagnani P., Franceschini S.S., Serio M., Ferrannini E. (2006). Interferon-gamma-inducible alpha-chemokine CXCL10 involvement in Graves’ ophthalmopathy: Modulation by peroxisome proliferator-activated receptor-gamma agonists. J. Clin. Endocrinol. Metab..

[75] Antonelli A., Ferrari S.M., Fallahi P., Frascerra S., Santini E., Franceschini S.S., Ferrannini E. (2009). Monokine induced by interferon gamma (IFNgamma) (CXCL9) and IFNgamma inducible T-cell alpha-chemoattractant (CXCL11) involvement in Graves’ disease and ophthalmopathy: Modulation by peroxisome proliferator-activated receptor- gamma agonists. J. Clin. Endocrinol. Metab..

[76] Antonelli A., Ferrari S.M., Frascerra S., Pupilli C., Mancusi C., Metelli M.R., Orlando C., Ferrannini E., Fallahi P. (2010). CXCL9 and CXCL11 chemokines modulation by peroxisome proliferator-activated receptor-alpha agonists secretion in Graves’ and normal thyrocytes. J. Clin. Endocrinol. Metab..

[77] Sahare P., Ruiz-Manriquez L.M., Anguiano B., Banerjee A., Pathak S., Duttaroy A.K., Luna-Bárcenas G., Paul S. (2025). Recent advances in nanomedicine for the diagnosis and therapy of thyroid disorders. 3 Biotech.

[78] Näreoja T., Rosenholm J.M., Lamminmäki U., Hänninen P.E. (2017). Super-sensitive time-resolved fluoroimmunoassay for thyroid-stimulating hormone utilizing europium(III) nanoparticle labels achieved by protein corona stabilization, short binding time, and serum preprocessing. Anal. Bioanal. Chem..

[79] Ibrahim N.H., Salimi M.N., Uda M.N.A., Parmin N.A., Hashim U., Afnan Uda M.N. (2020). New development quantification methods for salt iodine and urinary iodine using microfluidics based nanotechnology. IOP Conf. Ser. Mater. Sci. Eng..

[80] Kim S., Park J.W., Kim D., Kim D., Lee I.H., Jon S. (2009). Bioinspired colorimetric detection of calcium(II) ions in serum using calsequestrin-functionalized gold nanoparticles. Angew. Chem. Int. Ed..

[81] Sugamata Y., Uchiyama R., Honda T., Tanaka T., Matsunaga T., Yoshino T. (2013). Functional expression of thyroid-stimulating hormone receptor on nano-sized bacterial magnetic particles in Magnetospirillum magneticum AMB-1. Int. J. Mol. Sci..

[82] Kutlu Kaya C., Gümrükçü S., Saraç A.S., Kök F.N. (2020). A multifunctional long-term release system for treatment of hypothyroidism. J. Biomed. Mater. Res. A.

[83] Choi E.W., Shin I.S., Lee C.W., Youn H.Y. (2008). The effect of gene therapy using CTLA4Ig/silica-nanoparticles on canine experimental autoimmune thyroiditis. J. Gene Med..

[84] Chen K., Yang Y., Wu Y., Cao W., Zhao Y., Wang S., Wang K. (2025). PLGA nanoparticles encapsulating TSHR-A and rapamycin enhance the induction of dendritic cell-specific immune tolerance in mice with Graves’ disease. Biomed. Mater..

[85] Wang B., Yin Z., You X., Peng H., Jiang Y. (2025). Thyroid-Targeted Nano-Bombs Empower HIFU for Graves’ Disease. Adv. Sci..

[86] Wang B.W., Zhu R., Jin Y., Ouyang X., Jiang F.G., Wang X.H. (2025). Cerium oxide nanoparticles attenuates fibrosis and inflammation in thyroid-associated ophthalmopathy via JNK pathway. Front. Mol. Biosci..

[87] Han J.S., Kim S.E., Jin J.Q., Park N.R., Lee J.Y., Kim H.L., Lee S.B., Yang S.W., Lim D.J. (2021). Tear-Derived Exosome Proteins Are Increased in Patients with Thyroid Eye Disease. Int. J. Mol. Sci..

[88] Shi T.T., Zhao R.X., Xin Z., Hou Z.J., Wang H., Xie R.R., Li D.M., Yang J.K. (2022). Tear-derived exosomal biomarkers of Graves’ ophthalmopathy. Front. Immunol..

[89] Tan Y., Zhang F., Cao J., Feng L., Xie B., Gao L., Chen X., Yan Z., Xiong W. (2025). GPX1-driven selenium nanoplatform reprograms MAMs-mediated organelle crosstalk to reverse inflammatory adipose expansion in thyroid eye disease. Theranostics.

[90] Moog N.K., Entringer S., Heim C., Wadhwa P.D., Kathmann N., Buss C. (2017). Influence of maternal thyroid hormones during gestation on fetal brain development. Neuroscience.

[91] Brtko J., Podoba J., Macejova D. (2024). Selenium—Its role in physiology and endocrinology and as organoselenium compounds in oncology: A minireview. Endocr. Regul..

[92] Jolly J., Mohd Ahmar R., Zeeshan A. (2020). Selenium nanoparticles: Small is the new big: Mini review. Open J. Chem..

[93] Ansari J.A., Malik J.A., Ahmed S., Manzoor M., Ahemad N., Anwar S. (2024). Recent advances in the therapeutic applications of selenium nanoparticles. Mol. Biol. Rep..

[94] Leskela S., Serrano A., de la Fuente H., Rodríguez-Muñoz A., Ramos-Levi A., Sampedro-Nuñez M., Sánchez-Madrid F., González-Amaro R., Marazuela M. (2015). Graves’ disease is associated with a defective expression of the immune regulatory molecule galectin-9 in antigen-presenting dendritic cells. PLoS ONE.

[95] Leskela S., Rodríguez-Muñoz A., de la Fuente H., Figueroa-Vega N., Bonay P., Martín P., Serrano A., Sánchez-Madrid F., González-Amaro R., Marazuela M. (2013). Plasmacytoid dendritic cells in patients with autoimmune thyroid disease. J. Clin. Endocrinol. Metab..

[96] Pujol-Borrell R., Hanafusa T., Chiovato L., Bottazzo G.F. (1983). Lectin-induced expression of DR antigen on human cultured follicular thyroid cells. Nature.

[97] Aniszewski J.P., Valyasevi R.W., Bahn R.S. (2000). Relationship between disease duration and predominant orbital T cell subset in Graves’ ophthalmopathy. J. Clin. Endocrinol. Metab..

[98] Lee A.C.H., Kahaly G.J. (2020). Novel approaches for immunosuppression in Graves’ hyperthyroidism and associated orbitopathy. Eur. Thyroid. J..

[99] Pearce S.H.S., Dayan C., Wraith D.C., Barrell K., Olive N., Jansson L., Walker-Smith T., Carnegie C., Martin K.F., Boelaert K. (2019). Antigen-specific immunotherapy with thyrotropin receptor peptides in Graves’ hyperthyroidism: A phase I study. Thyroid..

[100] Codaccioni J.L., Orgiazzi J., Blanc P., Pugeat M., Roulier R., Carayon P. (1988). Lasting remissions in patients treated for Graves’ hyperthyroidism with propranolol alone: A pattern of spontaneous evolution of the disease. J. Clin. Endocrinol. Metab..

[101] Douglas R.S., Kahaly G.J., Patel A., Sile S., Thompson E.H.Z., Perdok R., Fleming J.C., Fowler B.T., Marcocci C., Marinò M. (2020). Teprotumumab for the Treatment of Active Thyroid Eye Disease. N. Engl. J. Med..

[102] Beretta M., Santos C.X., Molenaar C., Hafstad A.D., Miller C.C., Revazian A., Betteridge K., Schröder K., Streckfuß-Bömeke K., Doroshow J.H. (2020). Nox4 regulates InsP3 receptor-dependent Ca^2+^ release into mitochondria to promote cell survival. EMBO J..

[103] Arruda A.P., Pers B.M., Parlakgül G., Güney E., Inouye K., Hotamisligil G.S. (2014). Chronic enrichment of hepatic endoplasmic reticulum-mitochondria contact leads to mitochondrial dysfunction in obesity. Nat. Med..

[104] Missiroli S., Patergnani S., Caroccia N., Pedriali G., Perrone M., Previati M., Wieckowski M.R., Giorgi C. (2018). Mitochondria-associated membranes (MAMs) and inflammation. Cell Death Dis..

[105] Balkwill F., Mantovani A. (2001). Inflammation and cancer: Back to Virchow?. Lancet.

[106] Ferrari S.M., Fallahi P., Elia G., Ragusa F., Ruffilli I., Paparo S.R., Antonelli A. (2020). Thyroid autoimmune disorders and cancer. Semin. Cancer Biol..

[107] Elia G., Patrizio A., Ragusa F., Paparo S.R., Mazzi V., Balestri E., Botrini C., Rugani L., Benvenga S., Materazzi G. (2022). Molecular features of aggressive thyroid cancer. Front. Oncol..

[108] Gulwani D., Upadhyay P., Goel R., Sarangthem V., Singh T.D. (2024). Nanomedicine mediated thyroid cancer diagnosis and treatment: An approach from generalized to personalized medicine. Discov. Oncol..

[109] Xia T., Kovochich M., Liong M., Mädler L., Gilbert B., Shi H., Yeh J.I., Zink J.I., Nel A.E. (2008). Comparison of the mechanism of toxicity of zinc oxide and cerium oxide nanoparticles based on dissolution and oxidative stress properties. ACS Nano.

[110] Thi T.T.H., Suys E.J.A., Lee J.S., Nguyen D.H., Park K.D., Truong N.P. (2021). Lipid-Based Nanoparticles in the Clinic and Clinical Trials: From Cancer Nanomedicine to COVID-19 Vaccines. Vaccines.

[111] Dong Y., Siegwart D.J., Anderson D.G. (2019). Strategies, design, and chemistry in siRNA delivery systems. Adv. Drug Deliv. Rev..

[112] Barenholz Y. (2012). Doxil^®^—The first FDA-approved nano-drug: Lessons learned. J. Control. Release.

[113] Meyers P.A. (2009). Muramyl tripeptide (mifamurtide) for the treatment of osteosarcoma. Expert Rev. Anticancer Ther..

[114] Zong Y., Lin Y., Wei T., Cheng Q. (2023). Lipid nanoparticle (LNP) enables mRNA delivery for cancer therapy. Adv. Mater..

[115] Suhaimi N.A.A., Ahmad S., Husna S.M.N., Sarmiento M.E., Acosta A., Norazmi M.N., Ibrahim J., Mohamud R., Kadir R. (2022). Application of liposomes in the treatment of infectious diseases. Life Sci..

[116] Chaudhary N., Weissman D., Whitehead K.A. (2021). mRNA vaccines for infectious diseases: Principles, delivery and clinical translation. Nat. Rev. Drug Discov..

[117] Almenara-Fuentes L., Rodriguez-Fernandez S., Rosell-Mases E., Kachler K., You A., Salvado M., Andreev D., Steffen U., Bang H., Bozec A. (2023). A new platform for autoimmune diseases. Inducing tolerance with liposomes encapsulating autoantigens. Nanomedicine.

[118] Wang T., Suita Y., Miriyala S., Dean J., Tapinos N., Shen J. (2021). Advances in Lipid-Based Nanoparticles for Cancer Chemoimmunotherapy. Pharmaceutics.

